# Exploring bacteria diversity in commercialized table olive biofilms by metataxonomic and compositional data analysis

**DOI:** 10.1038/s41598-020-68305-7

**Published:** 2020-07-09

**Authors:** Antonio Benítez-Cabello, Verónica Romero-Gil, Eduardo Medina-Pradas, Antonio Garrido-Fernández, Francisco Noé Arroyo-López

**Affiliations:** 1 Food Technology Department, Instituto de La Grasa (CSIC), Crta Utrera km 1, Campus Universitario Pablo de Olavide. Building 46, 41013 Seville, Spain; 2 Technological Applications for Improvement of Quality and Safety in Foods, Carretera de Marbella nº22. Planta-1, 29108 Guaro, Málaga Spain

**Keywords:** Microbiology, Health care, Mathematics and computing

## Abstract

In this work, a total of 72 samples of non-thermally treated commercial table olives were obtained from different markets of the world. Then, prokaryotic diversity in olive biofilms was investigated by metataxonomic analysis. A total of 660 different OTUs were obtained, belonging to Archaea (2.12%) and Bacteria domains (97.88%). From these, 41 OTUs with a proportion of sequences ≥ 0.01% were studied by compositional data analysis. Only two genera were found in all samples, *Lactobacillus*, which was the predominant bacteria in the biofilm consortium (median 54.99%), and *Pediococcus* (26.09%). *Celerinatantimonas*, *Leuconostoc, Alkalibacterium, Pseudomonas, Marinilactibacillus*, *Weissella,* and the family *Enterobacteriaceae* were also present in at least 80% of samples. Regarding foodborne pathogens, only *Enterobacteriaceae, Vibrio,* and *Staphylococcus* were detected in at least 91.66%, 75.00%, and 54.10% of samples, respectively, but their median values were always below 0.15%. Compositional data analysis allowed discriminating between lye treated and natural olive samples, as well as between olives packaged in glass, PET and plastic bags. *Leuconostoc, Celerinatantimonas,* and *Alkalibacterium* were the bacteria genera with a higher discriminant power among samples. These results expand our knowledge of the bacteria diversity in olive biofilms, providing information about the sanitary and hygienic status of this ready-to-eat fermented vegetable.

## Introduction

The world's olive grove consists of more than 10 million hectares, of which over 1 million are destined to table olives, which constitute the most important fermented vegetable in the Mediterranean countries, with also noticeable productions in South America, USA and Australia. The last consolidated worldwide table olive balance shows that its consumption in 2018/2019 season was above 2.7 million tons^1^.

Among the various processing methods, alkali-treated olives (Spanish style), ripe olives by alkaline oxidation (Californian style) and directly brined olives (natural black or green olives) are the most common^2^. Besides, the industry makes diverse commercial presentations (mainly whole, pitted, and sliced) and use different packaging material (glass, PET, or bags, sometimes under vacuum) which lead to a great variety of products. Packaged table olives can be stabilized by pasteurization or sterilization, guaranteeing the absence of viable microbial cells. However, many times, they are preserved by their physic-chemical characteristics (pH, free acidity and salt) with or without use of preservatives. In these cases, the presence of microorganisms is usual^2^.

Because of the natural antimicrobial compounds of these fruits and the low pH and high salt levels reached during fermentation, table olives have a long history of microbial safety. However, in the absence of correct and hygienic handling practices, diverse microbial hazards may appear such us botulism, mycotoxins, biogenic amines or foodborne pathogens^3^. Thus, the study and control of the bacteria biodiversity during table olive processing can be useful for the evaluation and management of the safety risk associated with this ready-to-eat fermented vegetable, especially microorganisms with the ability to form biofilms on olive epidermis. Even so, most of the microbial studies on table olives have focused exclusively on the fermentative process, with scarce attention to the packaging and distribution phases^4–6^. Furthermore, the most-used approach has been based on culture-dependent techniques, which only allow counting and identifying viable cells^7^. However, the emergence of omic technologies and metataxonomic analysis represented a basic step for improving the knowledge of the microbial presence in foods, as they provide a broader vision of the existing biodiversity which could not be reached through the classical culture-dependent techniques. In table olives, various omic studies have been carried out to determine the bacterial and fungal evolution during the fermentation process^8–14^, but not in the finished products.

Metataxonomic analysis generates a considerable amount of information. However, standard multivariate statistical analyses may lead to, at least, formal incongruences when data are expressed in frequencies^15^. A first approach to analyse metataxonomic data could be Correspondence Analysis (CA)^16^. Metataxonomic results also represent a clear example of Compositional Data (CoDa), which are typically defined as vectors of positive components representing parts of a whole which carry relative information, usually with a constant sum (100 or 1). These conditions render most classical statistical techniques incoherent when applied to compositions, as such tools were devised for random variables in the Euclidean sampling space. The log-ratio approach was introduced to analyze CoDa data^17^. This solution was based on some log-ratio transformations of the original data, followed by the application of the standard techniques. The CoDa concept has already been applied in several fields, from genetic^18,19^ to the Spatial exploration^20^ among others. Its implementation in metagenomics could lead to more realistic segregation between samples than using standard multivariate methods. Recently, this approach was used to relate different inocula with the bacterial diversity found at the end of the Spanish-style green table olive fermentation^8^.

This work aimed to elucidate the bacteria composition in the biofilms of different commercial table olive samples through a metagenomic approach. Its relationship with the type of elaboration, presentation and packaging material/system, by applying CA and CoDa analysis, is also studied. The information could be useful to assess the hygienic and safety status of this important ready-to-eat fermented vegetable.

## Material and methods

### Sampling of olive packaging

A total of 72 commercial table olive samples were obtained from different supermarkets of Spain (n = 50), Greece (n = 6), France (n = 4), Chile (n = 4), Peru (n = 4), Portugal (n = 2), and Argentina (n = 2), between 2015 and 2017 years. None of them had undergone heat treatment such as pasteurization or sterilization for preservation, which was exclusively based on their physicochemical characteristics (pH, titratable acidity, and salt) or the use of authorized preservatives. The samples were analysed in the Food Biotechnology Department of Instituto de la Grasa (CSIC) within the first month of shelf life. They were classified according to the type of elaboration (SS: Spanish style n = 36, GN: green natural n = 26, or BN: black natural n = 10), presentation (P: pitted n = 12, W: whole n = 34, or S: sliced n = 26), and packaging material/system (P: PET = 28, B: bag n = 20, G: glass n = 18, or V: vacuum n = 6). They belonged to 16 different olive cultivars (*Manzanilla* n = 16, *Aloreña* n = 14, *Hojiblanca* n = 6, *Gordal* n = 2, *Morona* n = 2, *Verdial* n = 2, *Empeltre* n = 2, *Empeltre Mallorquina* n = 4, *Kalamata* n = 2, *Conservolea* n = 4, *Galega* n = 2, *Picholine* n = 2, *Criolla* n = 4, *Lucques du Languedoc* n = 3, *Azapa* n = 4, and *Arauco* n = 2). Table S1 (supplementary material) shows their references and origins.

### Physicochemical and microbiological analyses

The brine pH was measured using a Titroprocessor model 670 (METROHM, Switzerland). For determination of NaCl concentration, 0.5 mL of brine was mixed with 100 mL of distilled water. Then, titration of Cl^-^ was carried out with silver nitrate (AgNO_3_), using as the indicator a solution of potassium chromate (K_2_CrO_4_). The results were expressed as percentage (w/v) of NaCl.

For determining the viable bacteria populations on the olive surface, 25 g of fruits were washed with sterile saline solution (0.85% v/v) to remove no-adhered cells and pitted at sterile conditions. Then, fruit flesh was homogenised in a Stomacher (SEWARD LABORATORY SYSTEMS, Inc. Bohemia, NY, USA) for 2 min with 100 ml of sterile saline solution. Afterwards, direct or decimal dilutions were plated on selective culture media. Lactic acid bacteria (LAB) were plated on the Man, Rogosa and Sharpe (MRS) agar (OXOID, Basingstoke, Hampshire, England) supplemented with 0.02% sodium azide (SIGMA, St. Luis, USA), while *Enterobacteriaceae* was counted on Crystal-violet Neutral-Red bile glucose (VRBD) agar (MERCK, Darmstadt, Germany). Results were expressed as log_10_ CFU/g.

### DNA extraction from olive samples and sequencing

Twenty-five grams of olives from every sample were homogenized with 100 mL of sterile saline solution (0.9% NaCl) in a Stomacher homogenizer for 5 min, and the aqueous phase centrifuged at 9,000 × *g* for 15 min. In all cases, the supernatant was withdrawn, and the pellets were washed twice with sterile saline solution before stored at − 80 °C until use. Total genomic DNA from fruit samples was extracted and purified using the Power Food Microbial DNA Isolation Kit (MOBIO, Carlsbad, Calif) according to the manufacturer instructions, and sent for sequencing to FISABIO (Valencia, Spain). Before sequencing, the content of purified DNA was measured using a Qubit fluorometer (THERMO FISHER SCIENTIFIC, Waltham, USA), always obtaining values above 0.2 ng/µL.

The V3 and V4 region of the 16S ribosomal RNA gene was amplified^21^ following the 16S rDNA Gene Metagenomic Sequencing Library Preparation Illumina protocol (Part #1504423 Rev. A). Libraries were sequenced using a 2 × 300 pb paired-end run (MiSeq Reagent kit v3 (MS-102-3001) on a MiSeq Illumina platform, according to the manufacturer’s instructions. The quality evaluation of the sequencing was developed by the prinseq-lite program^22^ using a minimum sequence length of 50 bp, trim_qual_right of 30, triam_qual_type of mean and trim-qual_window of 20 bp. R1 and R2 read from sequencing were joined using the FLASH program^23^ by applying default parameters.

### Metataxonomic analysis

Metagenomic sequences in *.fna formats were labelled, concatenated and then analysed using the Quantitative Insights into Microbial Ecology (QIIME) pipeline (version 1.9.1), running Ubuntu v16.04. Sequences were first filtered by quality, excluding from downstream analyses those with a length out from 220 to 300 pb and a mean sequence Phred quality score < 35.

Chimeric sequences were identified and removed using Chimera slayer, and Operational Taxonomic Unit (OTU) tables were generated by using the UPGMA clustering method (Unweighted Pair Group Method with Arithmetic mean) and uclust to assign the OTUs^24^, through the script “Pick_de_novo_otus.py”. SILVA108 was used as a reference database^25^ defining OTUs at a level of ≥ 97% sequence homology. The Pick_de_novo_otus.py script generates an OTU mapping file, a representative set of sequences, a sequence alignment file, taxonomy assignment file, a filtered sequence alignment a phylogenetic tree, and a biom-formatted OTU table.

Cyanobacteria (which are confused with vegetable chloroplasts from olive cells), and mitochondria were excluded from the OTU tables using the script “filter_taxa_from_otu_table.py”. OTUs with a presence below 0.01% were also removed from the multivariate statistical analysis, but their frequencies are also shown in Table S2 (supplementary material).

The OTU tables were rarefied to 2,600 sequences (lowest number of reads obtained in one sample), and alpha diversity indexes (Chao, Observed Species, Shannon, Simpson, Good’s coverage) were calculated, using the Alpha_diversity.py script.

### Multivariate analysis

For each sample, the sum of all assigned OTUs was 100%. Therefore, for comparing associations containing counts of taxa, CA is an appropriate technique^16^. The objective of CA was to determine the similarities between the categories of samples and their populations of bacteria. This multivariate technique is more suitable when expecting that species have unimodal responses to the underlying parameters and become rare for lower parameters^16^. The analysis was performed using Past4 (Hammer et al., 2001) and the R package version^26^.

The microbiome data present a clear compositional structure and could be studied by CoDa^15,27^. Most of the CoDa analyses are based on log-ratio methodologies. Both ratios and logarithms are operations that require non-zero elements in the data matrix. As a consequence, any analysis of a vector of components should be preceded by a treatment of the zeros. For this purpose, CoDa considers three main types: rounded, count and essential^28^. The zeros in our data set match the first type, which applied when the value in the data matrix is not a true zero but rather represents an observed value below a particular rounding-off error or a very low value that cannot be recorded. The strategy to prevent the so-called zero- problem consists essentially of their replacement (imputation) by a small quantity. One of the most common is the multiplicative strategy. It simply consists of replacing each rounded zero in the data set by a small value (habitually 50–65% of the detection limit or the smaller value per variable) and, then, modifying the non-zero values in a multiplicative way^28^. In this work, the zeros were replaced by 65% of the lowest detected level for each variable. Then, diverse CoDa multivariate analyses could be applied. The biplot^29,30^ is a method which has been regularly applied to visualize the rows and columns of many different kinds of data matrices. The CoDa biplot was developed by Aitchison and Greenacre (2002)^31^. Two types of biplot are considered: covariance (rows in standard coordinates and columns in principal coordinates), which favour the display of the variables; and form (a reversed type of coordinates), which favour the display of individual. In both biplots, the row and column points are centred at the origin because of the double-centring transformation of the initial matrix. Other exploratory tools used were variation array and sequential binary partition (SBP)^28^. For the ANOVA performance and Discriminant Analysis (DA), the original data (in the Simplex) were subjected to an isometric log-ratio transformation (SBP or pivot coordinates) which transformed them into the Euclidean space, where the traditional statistical techniques can be applied. The statistical software used were: (i) CoDaPack, which is a stand-alone software developed by the University of Girone, Spain^32^, (ii) Package *compositions*^33^, and (iii) *robCompositions*^34^.

## Results

### Physicochemical and microbiological analysis

All table olive samples were analysed by microbiological and physicochemical methods (Table S1). The average pH value obtained for all of them was 3.94 ± 0.63, ranging from a minimum of 2.47 (S146 sample) to a maximum of 5.40 (S168). Depending of the type of elaboration, the average pH value was 4.05 ± 0.33 in GN, 3.96 ± 0.28 in BN, and 3.78 ± 0.81 in SS table olives. According to olive legislation^35^, the maximum pH value allowed for SS olives is 4.0 when fruits are preserved by refrigeration, addition of preservatives, modified atmospheres or by their own chemical characteristics. In the case of GN and BN olives, the pH value must be less than 4.3. On the contrary, the average salt concentration value was 5.97 ± 1.39%, ranging from a minimum of 3.28% (S139) to a maximum of 10.59% (S178). According to the type of elaboration, the average salt levels in brine were 5.50 ± 1.13% in SS, 6.17 ± 1.40% in GN, and 7.21 ± 1.53% in BN. In this case, the olive legislation^35^ establish a lower limit of 4.0% for SS olives preserved by refrigeration or by the addition of preservatives, while this limit must exceed 5.0% when SS olives are preserved by modified atmospheres or by their own chemical characteristics. In the case of GN and BN olives, the salt concentration must exceed 6%**.**

Because of the lack of thermal treatment, viable bacteria were present during shelf life. The average LAB population obtained for all treatments after the first month was 3.98 ± 1.97 log_10_ CFU/g, with certain samples (S104, S105, S140, S141, S146, S147, S148, S172, S173, S179) below the detection limit of the technique (< 1.60 log_10_ CFU/g) (see Table S1 supplementary material). The maximum LAB counts were obtained in SS samples, with an average value of 4.32 ± 1.82 log_10_ CFU/g, followed by GN with an average count of 3.77 ± 2.17 log_10_ CFU/g, and finally BN table olives with 3.38 ± 2.05 log_10_ CFU/g. On the contrary, *Enterobacteriaceae* population was always below the detection limit.

### Phylogenetic analysis

The massive sequencing of the 72 samples generated a total of 4,493,422 raw sequences using as a target the V3 and V4 domains of the 16S ribosomal RNA gene. After screening the data for poor-quality sequences, a total of 4,463,697 high-quality sequences were recovered and 3,731,003 assigned into OTUs. After removing chloroplasts and mitochondria, a total of 2,901,384 sequences (64.99% from the total of the raw sequences) were finally used for phylogenetic assignment, making a mean of 40,297 sequences per sample. S104 was the sample with the minimum number of sequences (2,623), while S159 had the maximum number (127,416 sequences). A total of 660 different prokaryotic OTUs were obtained, taking into consideration all table olive samples analysed. They belong to Archaea (phyla Euryarchaeota 2.12%) and Bacteria domains (main phyla reported: Proteobacteria 47.27%, Firmicutes 18.33%, Bacteroidetes 13.03%, and Actinobacteria 10.76%) (see Table S2 in supplementary material). An average of 198 OTUs per sample was obtained (Table S3, supplementary material), with GN and BN olives showing similar average values (172 and 178, respectively), and slightly higher for SS (average 221 OTUs per sample).

The diversity of the bacterial community was also analysed using rarefaction curves and richness estimator (Chao1 index). The Chao1 index varied from 100.28 (S126 sample) to 1,280.00 (S173). Prokaryotic biodiversity richness did not have a clear relation with any type of elaboration, presentation or packaging material/system. The average Chao1 indexes for GN, BN, and SS elaborations were very similar (613.78, 623.86, and 606.07, respectively). Overall, despite the diversity of sequencing depth between samples, the rarefaction analysis indicated that a number of bacterial reads above 2,600 per sample was enough. Thus, there was a satisfactory coverage of the bacteria diversity for all the samples analysed with Good's coverage values in many cases above 95% (see Table S3).

From the 660 prokaryotic OTUs detected, only 41 bacteria were found in at least one sample with a proportion of sequences ≥ 0.01% (Table 1). Two of them were present in all samples. *Lactobacillus* (B1) was the predominant genera ranging from a minimum of 4.32 (S105) to a maximum of 98.11% (S116) (median value 54.99%), followed of *Pediococcus* (B2) with a proportion of sequences ranging from 0.07 (S169) to 87.01% (S137) (median value 26.09%). The rests of OTUs were absent in at least one sample, but *Celerinatantimonas* (B9), *Leuconostoc* (B18), *Alkalibacterium* (B6), *Pseudomonas* (B12), *Marinilactibacillus* (B3), *Weissella* (B16), and the family *Enterobacteriaceae* (B14), were also present in at least 80% of table olive packages, albeit with median values in all cases below 0.5% (Table 1). As mentioned previously, Table S2 in supplementary material shows the frequency of sequences obtained for the 660 assigned OTUs (even when the frequency was lower 0.01%) in all samples.

**Table 1:** References of OTUs assigned at genera or family level (with a minimum percentage of sequences of 0.01% obtained in at least one sample) reported in this study.

OTU reference	OTU assignation	Frequency of samples with presence (%)	Median (%)	Maximum value (%)	Minimum value (%)
B1	*g. Lactobacillus*	100.00	54.99 (27.77)	98.11 (S116)	4.32 (S105)
B2	*g. Pediococcus*	100.00	26.09 (33.71)	87.01 (S137)	0.07(S169)
B3	*g. Marinilactibacillus*	84.72	0.11 (0.36)	19.01 (S120)	0.00
B4	*g. Vibrio*	75.00	0.11 (0.67)	11.58 (S131)	0.00
B5	*g. Halolactibacillus*	72.22	0.03 (0.08)	2.10 (S121)	0.00
B6	*g. Alkalibacterium*	86.11	0.13 (0.32)	41.27 (S166)	0.00
B7	*Unassigned*	100.00	0.44 (0.52)	3.48 (S105)	0.01 (S162)
B8	*F. Cardiobacteriaceae*	73.61	0.03 (0.12)	4.54 (S108)	0.00
B9	*g. Celerinatantimonas*	87.50	0.41 (2.89)	88.49 (S173)	0.00
B10	*g. Salinivibrio*	62.50	0.02 (0.06)	4.32 (S131)	0.00
B11	*g. Amphibacillus*	62.50	0.01 (0.08)	9.43 (S177)	0.00
B12	*g. Pseudomonas*	87.50	0.03 (0.07)	44.02 (S105)	0.00
B13	*g. Propionibacterium*	59.72	0.00 (0.01)	0.47 (S173)	0.00
B14	*F. Enterobacteriaceae*	91.66	0.05 (0.12)	21.39 (S105)	0.00
B15	*F. Bacillaceae*	51.38	0.00 (0.01)	0.44 (S151)	0.00
B16	*g. Weissella*	81.94	0.02 (0.06)	12.04 (S104)	0.00
B17	*g. Staphylococcus*	54.10	0.00 (0.01)	3.99 (S114)	0.00
B18	*g. Leuconostoc*	83.30	0.06 (0.17)	64.67 (S167)	0.00
B19	*g. Pantoea*	56.90	0.00 (0.01)	7.47 (S105)	0.00
B20	*g. Marinobacterium*	65.20	0.00 (0.02)	1.27 (S117)	0.00
B21	*g. Arthrobacter*	25.00	0.00 (0.00)	2.67 (S181)	0.00
B22	*g. Aequorivita*	6.94	0.00 (0.00)	1.51 (S180)	0.00
B23	*g. Chryseobacterium*	23.61	0.00 (0.00)	1.81 (S105)	0.00
B24	*g. Myroides*	20.83	0.00 (0.00)	1.36 (S127)	0.00
B25	*g. Paenibacillus*	16.67	0.00 (0.00)	1.76 (S105)	0.00
B26	*g. Oenococcus*	12.50	0.00 (0.00)	0.02 (S163)	0.00
B27	*F. Rhotobacteraceae*	41.66	0.00 (0.01)	5.08 (S114)	0.00
B28	*g. Rhodobacter*	15.27	0.00 (0.00)	1.74 (S181)	0.00
B29	*Uncultured*	33.33	0.00 (0.00)	1.39 (S180)	0.00
B30	*g. Novosphingobium*	34.72	0.00 (0.00)	2.29 (S105)	0.00
B31	*g. Sphingomonas*	40.27	0.00 (0.00)	2.18 (S105)	0.00
B32	*g. Helicobacter*	41.66	0.00 (0.00)	1.06 (S141)	0.00
B33	*g. Alteromonas*	59.72	0.00 (0.03)	0.15 (S188)	0.00
B34	*g. Marinobacter*	55.55	0.00 (0.02)	2.68 (S114)	0.00
B35	*g. Pseudoalteromonas*	38.89	0.00 (0.00)	0.11 (S127)	0.00
B36	*g. Enterobacter*	62.50	0.00 (0.02)	2.49 (S173)	0.00
B37	*g. Serratia*	55.55	0.00 (0.02)	2.24 (S105)	0.00
B38	*g. Cobetia*	34.72	0.00 (0.00)	0.02 (S149)	0.00
B39	*g. Halomonas*	64.11	0.00 (0.01)	1.17 (S180)	0.00
B40	*g. Marinomonas*	22.22	0.00 (0.00)	6.01 (S140)	0.00
B41	*g. Photobacterium*	16.67	0.00 (0.00)	8.39 (S140)	0.00

Percentage of samples with each OTU, median (interquartile range 25–75% in parenthesis), maximum, and minimum frequencies are also included.

**Figure 1:**
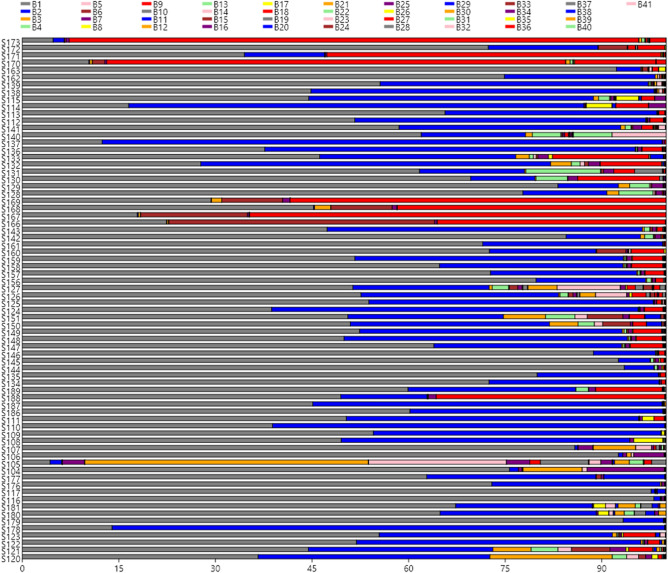
Abundance of the main OTUs**.** 41 OTUs (> 0.01% relative frequency) at genera and family level were found in the different table olive samples analysed in this work.

The distribution of the leading 41 OTUs in the 72 olive samples (Fig. 1) shows clearly that *Lactobacillus* (B1) was the most abundant genera in many packages (but not in S173, S170, S105 or S178) followed by *Pediococcus* (B2) which was, nevertheless, the most important in some others (S114, S137, S132, S124, S110, and S178). Also, *Celerinatantimonas* (B9) was abundant in several samples (S173, S171, S170, S169, S168, S167, and S189), while *Pseudomonas* (B12) and *Enterobacteriaceae* (B14) had an outstanding presence in S105. The other bacteria were found mostly in low proportion, except some samples (22.22% of the total) in which showed a complex mixture of genera (S115, S114, S137, S133, S132, S130, S126, S151, S150, S106, S105, S104, S180, S179, S121, and S120). Among the main 41 OTUs identified, the presence of microorganisms’ sequences with the potential to produce illness was reduced. *Enterobacteriaceae* (B14) was detected at least in 91.66% of samples, but with a median value of only 0.05%; *Vibrio* (B4) 75% of the samples, median value of 0.11%; and *Staphylococcus* (B17) 54% of samples, median value of 0.001%. The abundance of sequences of other foodborne pathogens was ever lower, detecting just in sporadic cases the presence of *Listeria, Legionella, Campylobacter, Clostridium, Yersinia, Escherichia* or *Salmonella* (Table S2, supplementary material)*.* Among spoilage microorganisms, only *Pseudomonas* (B12) (found in 87.50% of samples with a median value of 0.03%) and *Propionibacterium* (B13) (found in 59.72% of samples with a median value of 0.002%), were detected (Table 1).

### Correspondence analysis

**Figure 2:**
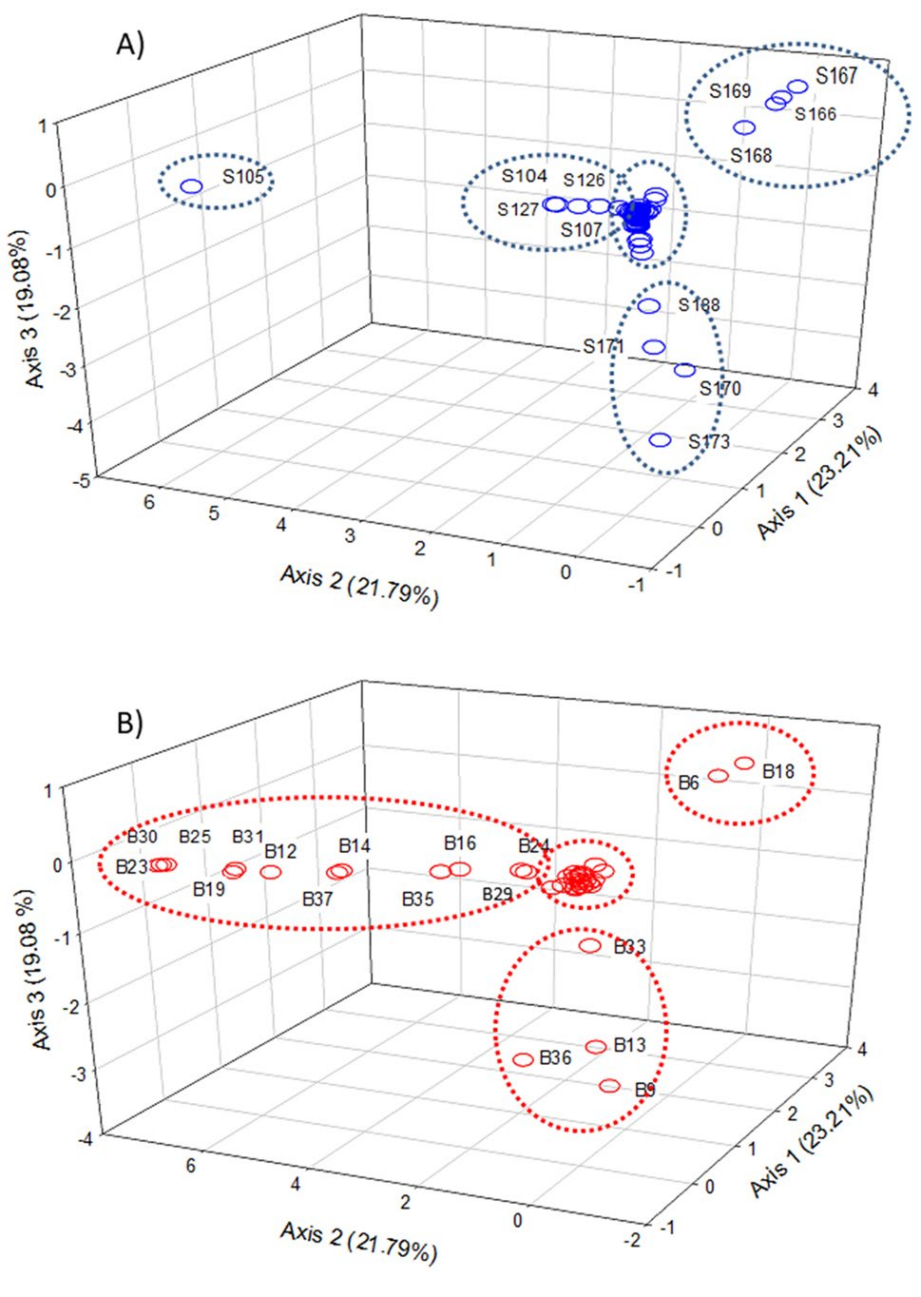
Study of similarity. Correspondence analysis among samples (**A**) and bacteria (**B**) was developed to study their similarity, and subsequent segregation of those different from the average.

A test of independence between rows and columns yielded a Chi-square of 148.268 (p < 0.0001), concluding that samples and bacteria were significantly associated. The percentage of inertia (variability) accounted by the first three axes was 23.21, 21.79, and 19.03, respectively, with a total of 64.08% explained inertia (Fig. 2). According to Fig. 2A, several table olive samples showed long distances from the mean. They were S166, S167, S168, and S169 along Axis 1 (all of them from *Picholine* and *Lucques du Languedoc* cultivars processed as SS), S105 along Axis 2 (*Aloreña* fruits processed as GN), and S170, S171, S173, and S188 samples along Axis 3 (*Empeltre* and *Criolla* cultivars processed as GN and BN, respectively). A few other samples were also relatively different from the majority group (S126, S127, S104, and S107, corresponding the two first to *Hojiblanca* olives processed as SS and the two later to *Aloreña* as GN olives).

According to the CA analysis, a total of 18 OTUs had discriminant power among samples. The most different OTUs profiles across samples (Fig. 2B) were *Chryseobacterium* (B23), *Paenibacillus* (B25), *Novosphingobium* (B30), *Pantoea* (B19), *Sphingomonas* (B31), *Pseudomonas* (B12), *Serratia* (B37), *Enterobacteriaceae* (B14), *Pseudoalteromonas* (B35), *Weissella* (B16), *Uncultured* (B29), and *Myroides* (B24) along Axis 2, *Alkalibacterium* (B6) and *Leuconostoc* (B18) along Axis 1, and *Celerinatantimonas* (B9), *Enterobacter (*B36), *Propionibacterium* (B13), and *Alteromonas* (B33) along Axis 3. Thus, comparing Fig. 2A,B, it is observed that the bacteria along Axis 1, *Alkalibacterium* and *Leuconostoc*, were more abundant in S166, S167, S168, and S169 samples. Similarly, *Chryseobacterium* (B23), *Paenibacillus* (B25), *Novosphingobium* (B30), *Pantoea* (B19), *Sphingomonas* (B31), *Pseudomonas* (B12), *Serratia* (B37), *Enterobacteriaceae* (B14), *Pseudoalteromonas* (B35)*, Weissella* (B16), *Uncultured* (B29), and *Myroides* (B24) were mainly detected in the sample segregated along the same axis (S105). Moreover, *Celerinatantimonas* (B9), *Enterobacter* (B36), *Propionibacterium* (B13)*,* and *Alteromonas* (B33) along Axis 3 would be characteristic of samples S170, S171, S173, and S188. The rest of 23 OTUs, including *Lactobacillus* (B1)*, Pediococcus* (B2)*, Marinilactibacillus* (B3)*, Vibrio* (B4)*,* and *Halolactibacillus* (B5)*,* which had a substantial presence in many samples, did not have discriminant power.

### CoDa analysis

**Figure 3:**
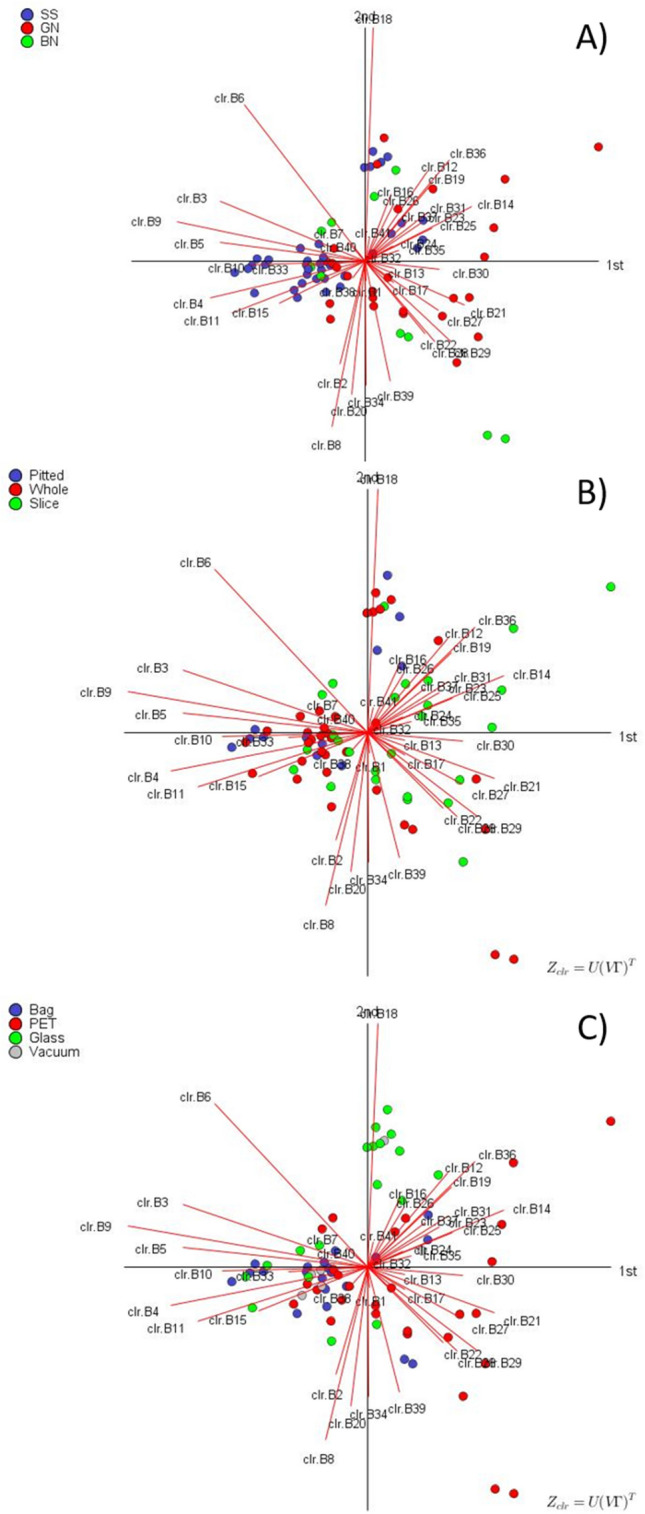
CoDa form biplot. Analysis done according to the type of elaboration (**A**), presentation (**B**), and packaging material (**C**), based on the proportion of each bacterium in the commercial samples analysed in this work.

The two principal components (PCs) of the CoDa biplot accounted for 39.36% of the total variance, which reached 49.62% when including the third PC. The characteristics and distribution of bacterial OTUs (regardless of grouping factor) were similar in covariance and form biplot (Fig. 3). Variables (bacteria) were mainly grouped along PC1, leading to large standard deviations (distance between two ends of rays) of their log-ratios. Besides, these log-ratios between the variables on both sides form small angles between them and, therefore, are strongly correlated. On the contrary, only a few variables were distributed along PC2: up (only *Leuconostoc*, B18) and down (*Pediococcus*, B2; *Cardiobacteriaceae*, B8; *Marinobacterium*, B20; *Marinobacter*, B34; and *Halomonas*, B39) and their log-ratios are also high but strongly correlated since their links are almost parallel. Also, as the angles between the links of the variables from the first and second groups are around 90º, their log-ratios are hardly related. Together, they contribute to the segregation of samples (PC1 along the horizontal axis and PC2 along the vertical). Table S4 (supplementary material) shows the variances of their log-ratios, which sum corresponds to the common variance of the *clr* transformed data. The largest values corresponded to *Celerinatantimonas* (clr B9) (10.89), *Leuconostoc* (clr B18) (8.54), and *Alkalibacterium* (clr B6) (7.39). The log-ratios of these variables eventually lead to the best segregation among samples. As the interest of this study was mainly focused on the difference between samples, the form biplot, according to the three factors involved in the design, is particularly appropriate: the distances in the plot approximate those between the samples (rows). Regarding elaboration, there is a trend for the segregation of SS from GN, although with some overlapping (Fig. 3A). A quite similar situation is observed for the type of presentation (Fig. 3B), with S olives situated on the right zone of the biplot and P on the left, but W distributed around the plot. In the case of the packaging material/system (Fig. 3C), those using P containers are mainly located on the right, B (together with V) on the left, and G spread from first to the third quadrant.

**Figure 4:**
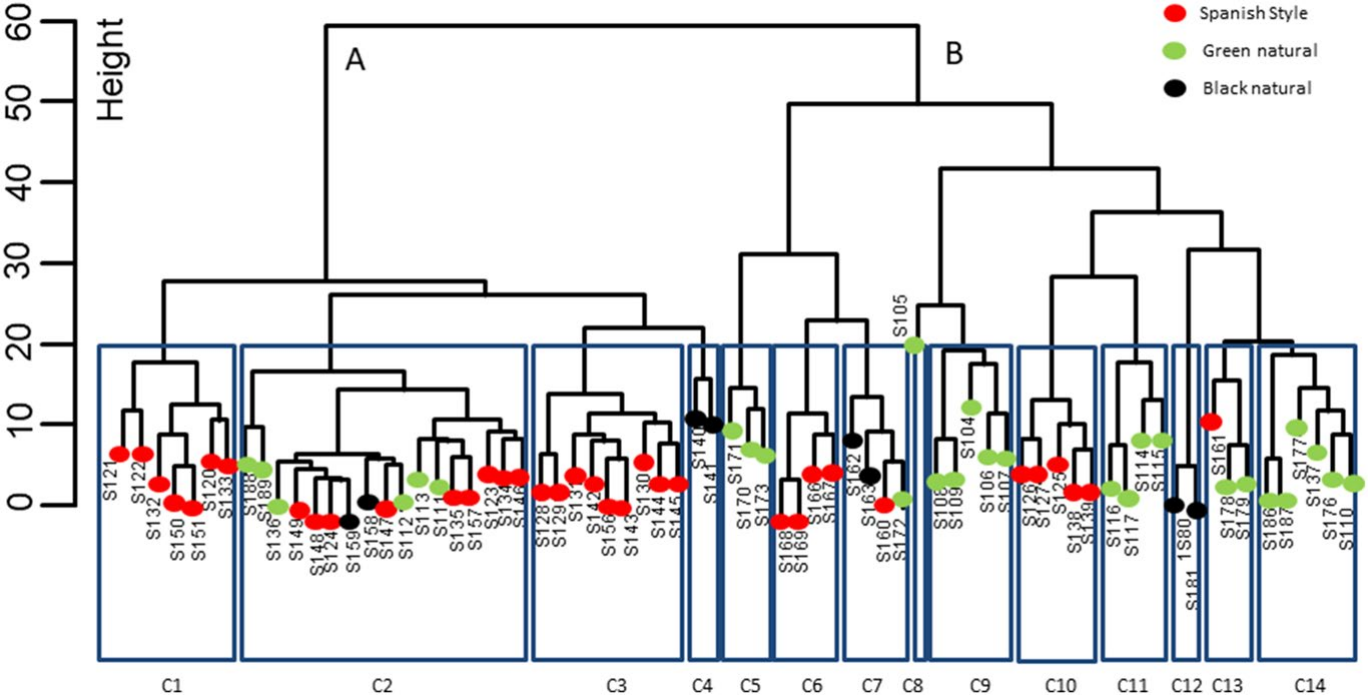
Cluster dendrogram of the samples. Dendrogram was done using the Ward.D2 on the pivot *coordinates* from the original data set. Heights of 60 and 20 were used for assessing dissimilarity.

When clustering analysis was applied (Fig. 4), considering the height of 60, two very different major groups were observed. Cluster A included 35 samples, many of them (71.42%) belonging to SS, while 28.58% were natural (GN or BN). Cluster B was composed of 37 samples, but, on the contrary, many of them (70.28%) were natural (GN or BN), and only 29.72% lye treated (SS). Hence, such dissimilarity allowed making gross discrimination between lye treated and natural olives. Considering a height of 20, a total of 14 different sub-clusters (C1-C14) were obtained. Notably, they included most of the samples from a similar origin in the same sub-cluster like S140 and S141 (cluster C4); S180 and S181 (C12); S170, S171, and 173 (C5). Nevertheless, some samples were singular and were not included in their analogous characteristic sub-clusters but in other groups of samples, like S105, S172, and S161 (C13) assigned to clusters C8, C7 and C13, respectively. Therefore, clustering was appropriate for grouping most of the samples from a similar type of elaboration and origins, but also displayed the presence of some others which bacteria consortium could not be related to the packaging conditions but other uncontrolled factors. These segregations are in agreement to the significant differences already observed for some groups in the CA (among others, S166, S167, S168, S169 (C6), S105 (C8), or S170, S171, S173 (C5).

CoDa ANOVA was also applied to evaluate the effect of elaboration, presentation and packaging material/system on bacteria diversity, using *ilr* transformed *coordinates*. Individually considered, the effect of type of elaboration was significant (at p = 0.0005165) while the effect of type of presentation (p = 0.1191) and packaging material (p = 0.0522) were not significant. However, the contribution of a new variable can be assessed by the additional variance explained by the model (already including the other two) when it is included. For this purpose, the multi-factor ANOVA, obtained by successively placing a different variable in the last position, was tested. In this case, the presentation form was not significant neither in the intermedia position or when it was added as the last term (see Table S5 in supplementary material). Therefore, the only two significant variables (factors) in the final ANOVA model were the type of elaboration and packaging material/system. Even so, despite the significant influence of both variables on the model, it only explained a reduced variance (≈15%), indicating that the bacterial diversity in the samples was, at least partially, randomly induced (or due to uncontrolled factors). As the model coefficients are in *ilr coordinates*, it was necessary the back-transformation of the ANOVA model predictions to present them in the original scale (Table S6, supplementary material). Regardless of elaboration, *Lactobacillus* (B1) would be the most abundant, with the highest level in the G containers, followed by its growth in P, V and B material/system, possibly associated with the permeability to oxygen of the different materials.

On the contrary, *Pediococcus* (B2), which is always expected, descends in the order B > V > P > G containers; that is, according to their decrease oxygen permeability. The presence of the remaining bacteria was not so clearly related to the packaging material. However, *Marinilactobacillus* (B3) was expected to be absent in olives packaged under vacuum (V), *Vibrio* (B4) and *Halolactibacillus* (B5) should be absent in P (GN) and V (GN) and P (GN), respectively. *Alkalibactyerium* (B6) could have a higher proportion in G (SS) and lower in the other materials. *Cardiobacteriaceae* (B8) was not present in G (SS) and B (BN), *Celerinatantimonas* (B9) would be absent from V (GN), *Leuconostoc* (B18) was not found in B (GN) or P (BN), S*alinivibrio* (B10) and *Amphibacillus* (B11) could mainly be related to SS while *Cardiobacteriaceae* with GN olives. As observed, overall, GN was more restrictive regarding biodiversity than the other elaborations.

**Figure 5:**
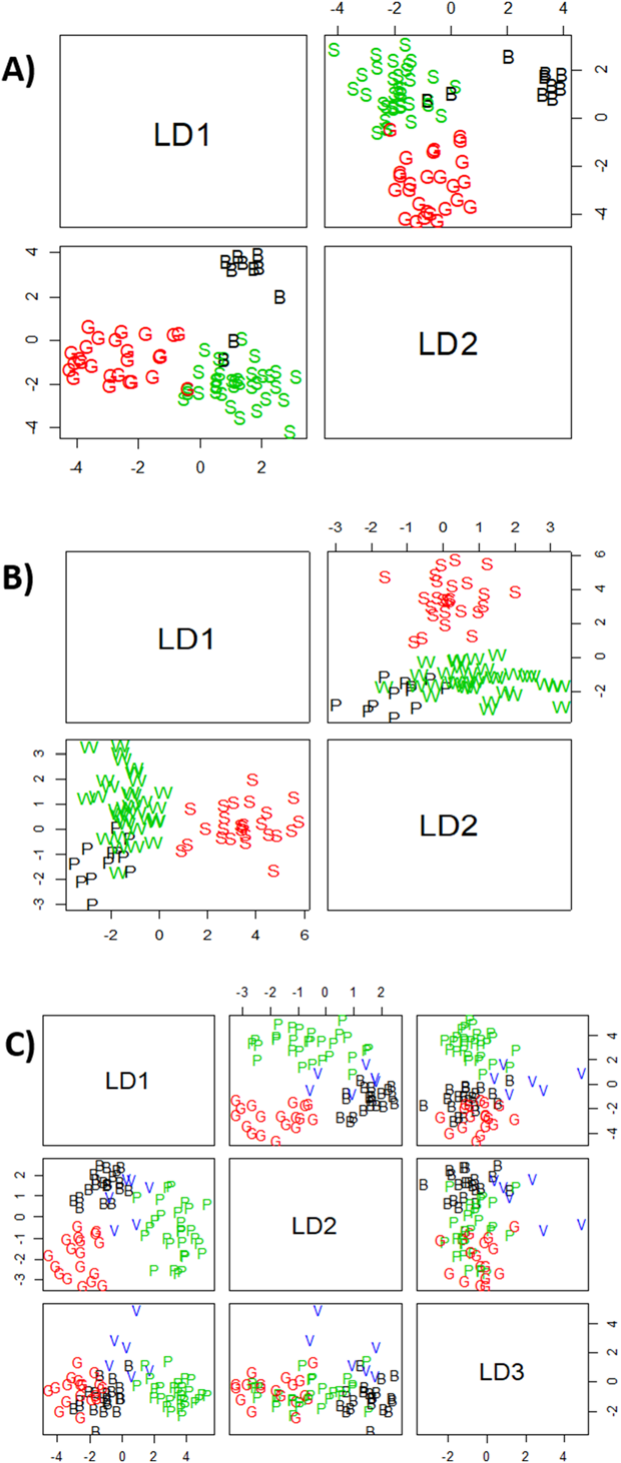
Segregation of the samples by CoDa linear discriminant analysis. Analysis was done according to elaboration (S-Spanish style, G-Green Natural, B-Black natural), presentation (S-Sliced, P-Pitted, W-Whole), and packaging material (G-Glass, P-PET, B-Bags, V-Vacuum).

LDA analysis was also performed on *ilr coordinates* (Euclidean space). There was an overall 95.83%, 94.44%, and 95.83% success in the classification according to the type of elaboration, presentation and packaging material, respectively (Table 2). Therefore, this supervised classical method provided fairly good classification. LDA was also applied using the package *compositions* (together with MASS for *lda*). This technique also provided the means of the different bacteria according to factors (elaboration, presentation, and packaging material/system). First in *ilr coordinates*, where mean and standard deviations are common parameters, and then as original values by back-transformation. The information (Table S7 in supplementary material) allows comparing the bacteria average according to levels of the factor co-variables. E.g., in elaboration, *Lactobacillus* (B1)*,* as an average, is present similarly in SS and GN in a proportion of ≈78% but in a slowly lower frequency in BN (≈72%). On the contrary, *Pediococcus* (B2) was more expected in BN (≈27%) than in GN (≈21%). Regarding the type of presentation, the differences were more limited for *Lactobacillus* (from approx. 75 to 79%) and approximately similar for *Pediococcus* (from 17 to 24%). On the contrary, in packaging material, the differences in the main two bacteria were sensible for both *Lactobacillus* (from 63 to 88%) and *Pediococcus* (from 8 to 35%). Situation that is in agreement with the significant differences observed among the levels of this factor in the ANOVA. The package *compositions* also works in *ilr coordinates* (Euclidean space) when performing LDA. The discriminant functions, for original values, is reproduced in Table S8 (supplementary material). This package showed similar segregation than *robComposition* but provided a visualization of the results. Two BN and one GN samples were wrongly classified as SS in case of the type of elaboration (Fig. 5a). S presentations were wholly differentiated from the rest of presentations, but several P samples (only two with robCompositions) were included in the W group (Fig. 5b), in agreement with the non-significant difference deduced in the ANOVA analysis. Regarding packaging material, based on the first two LDA functions, those using G and P containers were well segregated from that using B and V, but a few of V samples were wrongly classified as B (Fig. 5C), a situation compatible with the use plastic films in both samples. The discrimination according to packaging material using any other combination of LD functions did not improve the results since led to a similar or high degree of overlap between levels.

**Table 2:** Classification table of the LDA according to elaboration, presentation, and packaging material, using pivot *coordinates* and the classical option in the R package *robCompositions* (apparent success rate in parenthesis).

Elaboration (BN-Black natural, GN-Green natural, SS-Spanish style) (95.83%)				Presentation (P-Pitted, S-Sliced, W-Whole) (94.44%)				Packaging material (B-Bag, G-Glass, P-PET, V-Vacuum) (95.83%)				
	Predicted				Predicted				Predicted			
Actual	BN	GN	SS	Actual	P	S	W	Actual	B	G	P	V
BN	8	0	2	P	10	0	2	B	19	0	0	1
GN	0	25	1	S	0	26	0	G	0	18	0	0
SS	0	0	36	W	2	0	32	P	1	0	27	0
								V	1	0	0	5

## Discussion

Consumers increasingly demand healthier and safer foods. Table olives are an important component of the Mediterranean diet and culture, and as fermented vegetables, they are considered safe in a health perspective, provided that certain levels of pH and salt are obtained. The international legislation specifies the salt content and maximum pH limit for table olive packaging as a function of the type of elaboration^35^. In this study, albeit average values of pH (3.94) and salt (5.97%) were within the levels established by international legislation, 22% of the total samples were above the pH limit of 4.3 (close limit to germination of *Clostridium botulinum* spores). Such deviations might represent an obvious safety risk. However, *Clostridium* spp. were not detected in any table packaging analysed in this work above 0.01% of frequency. Some authors^36^, recently reported that a cocktail of diverse strains of *C. sporogenes* needed a pH value above 5.0 to germinate in both laboratory medium and table olive brine, which could explain why *Clostridium* was neither detected albeit pH value in certain samples was above 4.3. Our data are very similar to other studies on commercial table olives performed in markets of Spain^5^ and Portugal^6^, although with more than 12 years of difference. This way, while the first authors^5^ reported the average pH value of 3.9 and salt content of 5.3%, the second ones^6^ found average pH values of 4.0.

Regarding the microbial population in the products, the legislation only establishes that they must be free of any microorganisms, or their toxins, which could be a risk for consumer health^35^. A considerable number of the packages showed the presence of viable LAB populations (85% of samples) but, on the contrary, absence of *Enterobacteriaceae.* The average LAB population was 3.98 log_10_ CFU/g, with no statistical differences as a function of the type of elaboration. The presence of LAB populations in approximately 30% of Spanish products, reaching levels up to 7 log_10_ cfu/mL was also reported^5^. Others^4^ found that practically all the packages of lye treated olives showed microbial presence (ranging from 2 to 7 log_10_ cfu/ml). In natural olives, the percentage of samples with microorganisms was also high (78%). Moreover, some authors^6^ detected microbial presence in 64% of samples from table olives commercialized in Portugal. The presence of microorganisms habitually involved in the fermentation (LAB) in non-thermally treated olive packages could be considered usual, provided they do not produce changes that might compromise the safety of the product^2,35^.

As far as we know, this is the first study evaluating by molecular methods the prokaryotic diversity associated with olive biofilms from diverse international table olive markets. As above-commented, previous works have investigated, using culture-dependent methods, the microbial populations in the cover brines of commercial table olive packages from Italy^4^, Spain^5^, and Portugal^6^. However, microorganisms forming biofilms on fruit's epidermis could be ingested by the final consumers and depending on the bacteria consortium, had positive or negative connotations. Despite fermented table olives having a long history of microbial safety, different biological hazards (botulism, mycotoxins, biogenic amines and presence of foodborne pathogens) may be present in the finished products, usually due to improper olive handling practices^3^.

Metataxonomic analysis for the study of prokaryotic and eukaryotic microorganisms in table olive fermentation has been introduced recently^8–10,12–14,37^, but not in finished commercial products. The application of massive sequencing techniques to this fermented vegetable could help to assess the safety status of final products. This independent culture technique is based on the massive sequencing of total bacteria DNA obtained from olive biofilms, belonging to both alive and died cells. Results showed that the proportion of sequences of foodborne pathogens was in general very low (median value always obtained below 0.15%), being family *Enterobacteriaceae* and the genera *Vibrio* and *Staphylococcus* the undesirable microorganisms found in higher frequency. These data are in agreement with previous works^6^, which detected the presence of viable *Staphylococcus* in table olives commercialized in Portugal, and others, which did the same in seasoning material of natural green olives^38^. Furthermore, Benítez-Cabello et al. (2016)^39^ and Lucena-Padrós et al. (2014)^40^ detected, using molecular methods, *Vibrio* and *Staphylococcus* genera during industrial Spanish-style green table olive fermentation, while Abriouel et al. (2011)^41^ reported the presence of *Vibrio* spp. during the processing of natural green olives. *Vibrio* is a genus of halophilic Proteobacteria, which includes several species associated with human gastroenteritis diseases. *Staphylococcus* is a genus of bacteria very resistant to high salt, low pH (below 5.0) and fluctuating temperatures. Both genera include pathogenic and non-pathogenic species but, in any case, their relative presence should be taken into account as an indicator of the safety state of the final product. Finally, *Enterobacteriaceae* was always below the detection limit in all table olive samples analysed in this work, albeit DNA sequences for this bacteria family were detected in 91.66% of samples at low proportions (except in S105 sample with 21.39%). The presence of *Enterobacteriaceae* during table olive processing is frequent, but they are inhibited at the usual low pH levels that are reached during table olive packaging^2^. Our data are also in agreement with other authors^5^, who did not report viable *Enterobacteriaceae* presence in commercial Spanish cultivar table olives.

Bacteria and Archaea are the most ancient and most widespread forms of life on Earth. In this work, a total of 660 different prokaryotic OTUs (97.9% bacteria) were obtained when analysing the 72 olive packages, but only 41 OTUs were a majority and had frequencies > 0.01% in at least one sample. However, our biodiversity indexes were higher than those obtained in other metataxonomics studies performed during table olive fermentations^12,13,37^. These authors reported the presence of OTUs at family/genera family/genera level in many cases below 100. On the contrary, in the study carried out on the seasoning material (salt, aromatic herbs, fermented garlic, or pepper) used during table olive packaging, a higher number of OTUs (> 3.000) were found^42^. This abundance could explain the intermediate values between fermented fruits and seasoning material found in this survey because, in olive packages, diverse types of seasoning material are added. It was reported using several statistical approaches and analysing > 700.000 prokaryotic 16S-V4 sequences, that could exist about 2.2–4.3 million bacterial OTUs worldwide, refuting recent predictions of trillions of prokaryotic OTUs in the biosphere^43^. According to their data, the bacterial biodiversity in table olive packaging could stand for 0.015% of the worldwide prokaryotic diversity.

CoDa analysis identified *Lactobacillus* and *Pediococcus* as the ubiquitous bacteria in the packages of this fermented vegetable, widely distributed around the world. Both genera are usually isolated from diverse types of table olive fermentations and be responsible for its lactic acid process^2,44^. This information reinforces the healthy and nutritional aspects of this fermented food, proving that table olives in a splendid carrier of potential probiotic *Lactobacillus* strains to final consumers. In this sense, *L. pentosus LPG1* isolated from table olive biofilms recently proved its high probiotic potential in mammals^45^. Furthermore, the work has demonstrated that following methodologies developed for CoDa, as the proportion of OTUs, the statistical analysis can lead to results that allow testing the different hypothesis of the experiment while working in the suitable sampling space. Therefore, the work might also be considered as a query for the appropriate study of metagenomic information, as was previously reported^8^.

## Conclusion

This work has proven the effectiveness of the application of metagenomics and multivariate analysis based in CoDa to expand our knowledge of the prokaryotic diversity in olive biofilms. Results have shown the absence of food-borne pathogens in many samples, but suggesting to *Vibrio, Staphylococcus,* and *Enterobacteriaceae* as potential biomarkers of the safety and hygienic status of this ready-to-eat fermented vegetable. Moreover, the CoDa exploratory techniques presented the distribution map of samples and discrimination as a function of elaboration (lye treated *vs* natural olives) and packaging material (among glass, PET, and bags). *Lactobacillus* and *Pediococcus* were the most widely distributed and ubiquitous bacteria in all the samples. At the same time, the presence of other genera like *Propionibacterium, Pantoea, Marinobacterium*, *Alteromonas* or *Marinobacter* could be considered as sporadic and randomly distributed. However, further studies are necessary to corroborate the microbial activity of all these microorganisms after packaging, because microbial DNA could belong to viable or died cells.

## Supplementary information

Supplementary file1 (XLSX 270 kb)
